# Navigating the fulfillment of needs in university students: parenting styles, body perception, and well-being

**DOI:** 10.3389/fpsyg.2025.1698341

**Published:** 2025-12-15

**Authors:** Giulio D’Urso, Luana La Marca, Rossella Marzullo

**Affiliations:** Mediterranean University of Reggio Calabria, Reggio Calabria, Italy

**Keywords:** university students, wellbeing, parenting style, body perception, needs

## Abstract

The literature has highlighted the importance of investigating the factors that influence the satisfaction of university students’ basic psychological needs for relatedness, competence, and autonomy. This study, indeed, aims to examine whether parenting style can affect students’ general well-being and attitudes toward their body, and whether these factors, in turn, influence the fulfillment of their basic psychological needs. The sample consisted of 189 university students (177 females and 12 males) who completed a series of self-report questionnaires measuring perceived parenting style (Perceptions of Parents Scales, POPS), body image (Body Appreciation Scale-2, BAS-2), general well-being (Mental Health Continuum–Short Form, MHC–SF), and need satisfaction (Basic Psychological Satisfaction and Frustration Scale, BPNSF). Path analysis revealed that a positive parenting style has a significant positive impact on both body image and overall well-being. Among these two factors, only general well-being significantly predicted the satisfaction of basic psychological needs. Theoretical and clinical implications are discussed.

## 1 Introduction

The satisfaction of university students’ basic psychological needs — such as relatedness, autonomy, and competence — can be supported by a positive perception of one’s own body (Brichacek et al., 2018; Möri et al., 2022) and a high level of general well-being (Li et al., 2025). These factors, in turn, may be influenced by socio-emotional variables, particularly family harmony, which serves as a significant source of support in students’ developmental and educational pathways (Schnettler et al., 2017). The satisfaction of such psychological needs, more generally, represents a key developmental task for young adults. If these needs are not adequately met, individuals may face future risks and may fail to successfully complete—or may inadequately fulfill—the developmental tasks associated with later stages of the life cycle (Ryan and Deci, 2000).

Specifically, the literature highlights that a key factor for the mental well-being of students embarking on their university journey is parenting style (El-Ghareap Hassan et al., 2025), which continues to play an important role during this developmental phase (Huang et al., 2024), especially in the Italian cultural context (Olivari et al., 2015). Several strands of research emphasize how supportive parenting styles, which promote self-esteem and emotional well-being, serve as a crucial protective factor for improving the mental health of adolescents or young adults (Peng et al., 2021; Azman et al., 2021), particularly when they are university students (e.g., Liu et al., 2024). In line with Olson’s circumplex model (e.g., Olson et al., 2019), emotionally organized families capable of establishing healthy trust relationships and setting appropriate boundaries with their children can enhance emotional and social abilities, foster well-being and preventing entry into vicious cycles of psychopathology or psychopathological traits. Furthermore, parenting styles aimed at promoting self-esteem and personal worth can be especially important, as when an adolescent or young adult—particularly one engaged in an academic path—improves their overall well-being, they become more resilient and better equipped to develop their identity (Kou, 2022). This is particularly crucial during young adulthood, as it can help prevent risky emotional and behavioral behaviors (Jackson et al., 2012), which may undermine engagement (Symonds et al., 2023).

Additionally, another aspect of parenting style, particularly during the transition from adolescence to young adulthood, concerns body perception (Pace et al., 2018; Rodgers and Chabrol, 2009). During this developmental phase, indeed, the body’s shape is consolidated, and young adults face possible negative attitudes toward their bodies (Yang et al., 2025; Khosravi et al., 2023), especially under the influence of a society increasingly driven by perfect body models, even though there is a growing movement toward promoting a body that is not perfectly aligned with the “perfect” body standards as the sole solution for acceptance (Aparicio-Martínez et al., 2017). A parenting style that promotes self-esteem and, more broadly, emotionally supports their child can represent a safe space where the child can experiment with themselves without experiencing obstructive or hostile behaviors, even though the social fabric often promotes unrealistic and unattainable body models (Pritchard and Button, 2024). Thus, a positive parenting style can impact emotional stress management when the adolescents reflect on their body and the perceptions related to it. If they feel accepted by their parents first, they can experiment with and internalize a positive self-image and, above all, a “valid bodily self” in every circumstance (Szkody et al., 2021; Izydorczyk et al., 2021).

However, at the same time the literature highlights how positive attitudes toward the body combined with well-being or mental health may represent factors capable of satisfying autonomy, relational, and competence needs (Sakellariou, 2023; Lôbo et al., 2020; Ferreira-Barbosa et al., 2024). In other words, a positive body image during young adulthood may serve as a resource for the development of a solid, rather than diffuse, identity, enabling the self to recognize its needs and adequately satisfy them, by drawing on the positive perception of body-related aspects of identity (Andrew et al., 2016). At the same time, general well-being acts as an emotional regulator capable of leading the young adult toward the fulfillment of relational, autonomy, and competence needs (e.g., De-Juanas et al., 2020; Melendro et al., 2020; Khan et al., 2024). By experiencing a state of well-being, they are more motivated to learn, stay engaged, face challenges, and develop new competencies, as they feel capable of doing so (Chen et al., 2023), freely choosing and developing authentic connections (Ryan and Deci, 2000). In line with this literature, this study aims to test whether, in a group of university students, parenting style can influence well-being and attitudes toward the body, and whether these can consequently impact the fulfillment of fundamental needs during this phase of the life cycle, in line with the developmental cascade perspective (Masten and Cicchetti, 2010). According to the authors, experiences during adolescence, including parental influence, can trigger a series of events that extend over time, influencing various domains of development, including body identity and well-being, causing cascading reactions that shape psychological and social development throughout life, creating opportunities or obstacles for satisfying needs.

## 2 Materials and methods

### 2.1 Participants and procedure

The sample consisted of 189 university Italian students (177 females and 12 males) aged 18 to 48 years (*M* = 23.7; SD = 5.93), mostly enrolled in degree course of human sciences (95.24%). Participants were recruited between March 2025 and June 2025 via academic lectures and through words of mouth at the Mediterranea University of Reggio Calabria. Participants anonymously completed an online survey including self-report measures on perceived parenting style, body image, general well-being and need satisfaction. Sociodemographic variables were also collected. Statistical analyses were undertaken to explore the relationships between the investigated variables, and to test the hypotheses of the current study. All procedures were performed in accordance with the ethical standards of the institutional and/or national research committee, and with the 1964 Helsinki declaration and its later amendments or comparable ethical standards.

### 2.2 Measures

*Perceptions of Parents Scales* (POPS; Grolnick et al., 1997) is a set of self-report instruments that assess children’s and college students’ perceptions of their parents’ autonomy support, involvement, and warmth. Rooted in Self-Determination Theory (SDT), the scales measure how well parents provide an optimal parenting context to foster a child’s autonomy, competence, and motivation. Examples of items from the Perceptions of Parents Scale (POPS) include statements like “My father/mother allows me to decide things for myself,” “My father/mother finds time to talk with me” and “My mother/father seems to be happy to see me.” In this study we selected six items of the College-Student Scale and calculated mean score to gather insights into the quality of parent–child relationships and their impact on well-being. Psychometric properties vary by specific version and sample, but research generally indicates strong reliability (internal consistency) and validity, with factor analyses confirming the proposed underlying dimensions of parenting. Internal consistency of the scale in the current study was good (Cronbach’s alpha = 0.88).

*Body Appreciation Scale-2* (BAS-2; Tylka and Wood-Barcalow, 2015) is a 10-item, 5-point Likert scale that measures a positive body image by assessing an individual’s acceptance, respect, and positive opinions toward their own body, irrespective of societal beauty standards. The scale assesses appreciation for the body’s functionality and uniqueness, responses are summed to create a total score, with higher scores indicating greater body appreciation. Examples of items include statements like “I feel good about my body” or “I feel that my body has at least some good qualities.” The BAS-2 is a psychometrically sound, unidimensional measure of positive body image with good internal consistency and test–retest reliability. It has proven to be applicable across various cultures and demographic groups, with its factor structure and validity consistently supported in different national contexts and languages. Cronbach’s alpha of the BAS-2 in the present study was an excellent 0.97.

*Mental Health Continuum-Short Form* (MHC-SF, Keyes, 2007) is a 14-item self-report questionnaire that measures positive mental health across three dimensions: emotional, psychological, and social well-being. An example item is: “How often did you feel interested in life?” Other examples include items about feeling “happy,” “satisfied with life,” and feeling “confident.” These items assess emotional, psychological, and social well-being over the past month using a six-point (0 = Never, 5 = Every Day) frequency scale. Scores are used to classify individuals as flourishing, moderately mentally healthy, or languishing, providing a continuous measure of well-being. A higher total score indicates a higher level of overall mental well-being. The total score can also be broken down into scores for three subscales: Emotional Well-being, Social Well-being, and Psychological Well-being. The MHC-SF generally demonstrates good psychometric properties, including high internal consistency (Cronbach’s alpha is often >0.90 for the total scale) and construct validity through strong correlations with related measures of well-being and mental health. The MHC-SF has shown reliability and validity in various populations and contexts, making it a psychometrically sound instrument for measuring positive mental health. Internal consistency of the scale in the current study was very good (Cronbach’s alpha = 0.91).

*Basic Psychological Satisfaction and Frustration Scale* (BPNSF; Chen et al., 2015) is a self-report questionnaire to assess both the fulfillment and thwarting of the three basic psychological needs central to Self-Determination Theory: autonomy (feeling in control of one’s actions), competence (feeling effective and capable), and relatedness (feeling connected to others). We adopted 12 items (the scale typically features 24 items), divided into six subscales (autonomy satisfaction and frustration, competence satisfaction and frustration, and relatedness satisfaction and frustration). An example item is “I feel pressured to do many of the things I do at work” from the Autonomy Frustration subscale, or “I feel confident that I can do things well” from the Competence Satisfaction subscale. Items are typically rated on a 5-point scale (1 = Not at all to 5 = A lot), in this study mean score was calculated, so higher scores indicate greater satisfaction or greater frustration for that specific need. It has been widely translated and validated across various cultures and contexts, research confirms its good internal consistency and reliability. Internal consistency of the scale in the current study was good (Cronbach’s alpha = 0.80).

### 2.3 Analysis plan

Correlational analyses were conducted between the variables under study. Moreover, a path analysis model was conducted using Jamovi to examine the relationships between perceived parenting style, in conjunction with general well-being and body image, and their associations with need satisfaction. Finally, the study aims to investigate whether these two outcomes are connected to the basic psychological needs of autonomy, competence and relatedness.

## 3 Results

The intercorrelations between the investigated variables are presented in Table 1.

**Table 1:** Correlation among variables.

	1.	2.	3.	4.
1. POPS – Parenting style	/	0.32***	0.49***	0.34***
2. BAS-2 – Body image		/	0.57***	0.23**
3. MHC-SF – Well-being			/	0.51***
4. BPNSF – Need satisfaction				/

Pearson’s *r* correlations *** = *p* < 0.001 ** *p* < 0.01.

**Figure 1:**
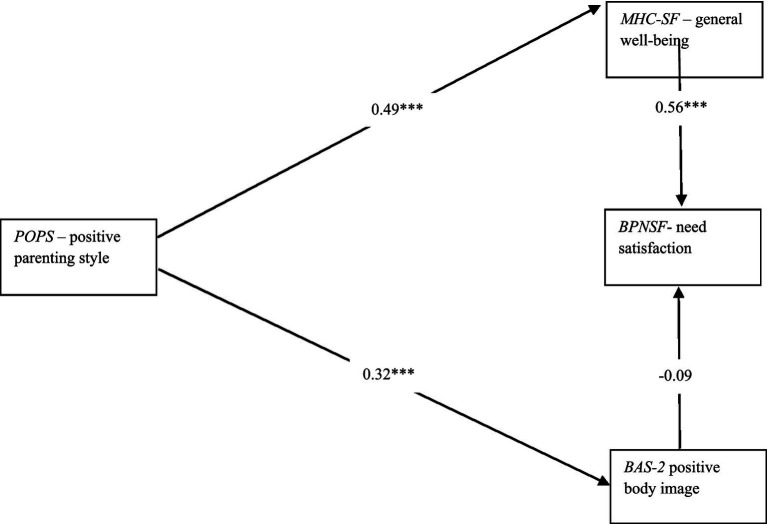
Summary model. R^2^_(general well-being)_ = 0.24, R^2^_(positive body image)_ = 0.11, R^2^_(need satisfaction)_ = 0.30.

Model fit statistics indicate that the path analysis model fits the data well, with a root mean square error of approximation (RMSEA) of 0.3 with *p* < 0.001, and a comparative fit index (CFI) of 0.71. The model reveals that positive parenting style positively predicts general well-being (*b* = 0.49; *p* < 0.001) and positive body image (*b* = 0.32; *p* < 0.001). Moreover, general well-being positively predicts need satisfaction (*b* = 0.56; *p* < 0.001) but on the other hand, positive body image does not predict need satisfaction (*b* = −0.09; p n.s.). Model is shown in Figure 1.

The overall model fit indices (specifically CFI and RMSEA) do not reach the levels commonly considered highly satisfactory in the literature. This outcome can be attributed to several structural factors. First, the model is relatively simple, with a limited number of variables and few degrees of freedom—a characteristic that tends to penalize incremental indices such as CFI (Bentler, 1990). Second, although the sample is adequate, it cannot be considered particularly large or highly heterogeneous, a condition that may negatively affect the stability of the estimates and the corresponding fit indices. Finally, it is possible that the model includes nonlinear relationships or correlated residuals that were not explicitly modeled, elements that may contribute to deteriorating the global fit indices. Despite this, the variance-based indices (R^2^) show that the variables significantly predict the considered outcomes, with values ranging from 0.11 to 0.30, all statistically significant (*p* < 0.001). This suggests that, even in the presence of a suboptimal global fit, the model retains a good predictive capacity, making the results fully interpretable and substantively meaningful. Moreover, the literature indicates that RMSEA tends to be overvalued in models with few degrees of freedom, making it at times a not entirely reliable indicator under such conditions (Kenny et al., 2015). It is important to emphasize that the primary aim of the analysis was not to optimize the global model fit, but rather to verify specific theoretical relationships, all of which proved significant and consistent with the predefined hypotheses.

## 4 Discussion and conclusion

The present study sought to examine, within the framework of the developmental cascade perspective, whether parenting styles exert an influence on body image and overall well-being, and whether these, in turn, cascade to affect the satisfaction of developmental needs characteristic of this life stage in the student population (e.g., relational needs, autonomy, and competence). The findings revealed that positive parenting styles significantly contribute to the promotion of a positive body image as well as to general well-being. In line with the literature (Yang et al., 2025; Khosravi et al., 2023), parenting practices oriented toward fostering self-esteem and autonomy were found to facilitate the development of identity, and specifically a stable and coherent sense of self. This sense of self, being less reliant on external standards or social comparisons, is consequently more resilient to distorted socio-cultural messages. Parental encouragement of independence thus creates an affective climate that validates a positive self-image in young adults, enabling them to cultivate self-respect and to develop an adequate acceptance of, and confidence in, their own bodies.

Furthermore, according with Olson’s model (Olson et al., 2019) positive parenting was shown to enhance general well-being by supporting the establishment of clear, non-enmeshed family boundaries, which foster autonomy and self-care while mitigating the risk of maladaptive or psychopathological traits. Through such caregiving practices, young adults are able to internalize interactive models that promote self-confidence and equip them to address developmental challenges, including those pertaining to academic trajectories (Zahra and Saleem, 2021). Importantly, results indicate that, within the cascade process, it is general well-being alone that exerts a direct effect on the satisfaction of relational, autonomy, and competence needs (Martela and Sheldon, 2019). This outcome may be explained by the fact that young adults who have internalized positive socio-emotional schemas transmitted by parental figures possess resources and secure-base models that prevent destabilization. Such a condition of global well-being thus represents a pivotal resource, rendering them better equipped to confront the demands inherent in the transition to adulthood.

Having established the connection between appropriate parenting and greater psychophysical well-being in both boys and girls, it is important to emphasize—particularly from an educational perspective—that the individual is inevitably part of a transpersonal field (Foulkes, 1991, 2012). Within this field, communities, families, and individuals intersect in a continuous interplay of historical legacy and current lived experience (Riva, 2021). This social, historical, and educational transpersonal field includes the family transpersonal field, where significant emphasis is placed on the couple from whom the person descends. This couple is characterized by a particular style of shared living, which, consciously or unconsciously, establishes a specific educational approach to the care, nurturing, and development of the children.

The youngadult-as-subject thus emerges as the result of a confluence of educational experiences, traditions, transgenerational loyalties, legacies, and a self-formative drive toward emancipatory autonomy. Despite its historical variability, the family continues to function as an archetypal reference system for values. As Parsons (1996) notes, it operates as a genuine “workshop” for the production of human personality. Our study suggests that the family context can indeed foster emancipatory impulses. From this perspective, an interdisciplinary approach—combining pedagogy, psychology, and other intersecting disciplines—can offer valuable insights into the methods and practices by which individuals are educated and shaped within their environments. This process involves the internalization of experiences and meanings gained through intersubjective relationships and the broader socio-historical context, starting with the family or families to which one belongs. In the contemporary landscape, this includes diverse and evolving family structures across the lifespan. Simultaneously, the insights derived from data analysis can contribute to the development of theoretical and methodological frameworks aimed at designing educational and training programs. These programs would support individuals in re-experiencing and processing early educational experiences from birth onward (Cigoli, 2013; Riva, 2004). Furthermore, the study of social phenomena must always be integrated with the analysis of subjective meanings of action (Weber, 1961), as motivational aspects are essential to social, pedagogical, and psychological research. Such research should be guided by the paradigm of complexity, as proposed by Morin (2001), in order to move beyond reductive and oversimplified approaches—approaches which are increasingly inadequate even in addressing the lived realities and responses of children. Morin (2001) emphasizes that love is the most complex communicative experience that life has ever generated. This insight underscores why appropriate parenting styles can generate a sustained state of well-being that positively impacts multiple spheres of human existence.

The finding that only general well-being, rather than positive body perception, is associated with the fulfillment of basic psychological needs (autonomy, competence, and relatedness) may be explained by the broader nature of well-being as a construct, encompassing multiple dimensions—physical, emotional, relational, and motivational (Maddux, 2017; Oishi and Diener, 2001). At the same time, it is plausible that body- related concerns represent an aspect that has already reached relative stabilization and, therefore, holds less relevance for the present sample, given that participants have already completed the developmental transition from adolescence to young adulthood. In addition, this last result (i.e., that body image does not predict general well-being) from a clinical point of view could suggest the need to go beyond the supremacy of the body and appearances to feel good and be satisfied.

Body perception is strongly influenced by social norms, cultural pressures, and group dynamics, its impact on basic psychological need satisfaction may vary across contexts. It is possible that this association is more pronounced during adolescence and gradually diminishes in young adulthood, as may be the case in our sample, especially when overall well-being becomes a more central concern than body-related issues. Young adults may also typically develop more advanced self-regulation skills and greater cognitive maturity, which can buffer the emotional impact of body-related concerns, as their focus shifts toward overall well-being and identifying aspects of themselves that reinforce a positive sense of identity (e.g., Vangeel et al., 2018).

In this sense, “going beyond the body and its appearance” could mean focusing on what goes beyond physical appearance to understand and value one’s (and others’) whole person, promoting deeper and more authentic connections with both oneself and others. This process involves developing greater awareness of one’s mental processes, balancing social expectations with one’s inner identity, and cultivating qualities such as personality and empathy to build meaningful relationships (Tsakiris, 2017). This finding has also a significant impact on social and health policies, guiding wellness promotion programs aimed at young people, such as the establishment and funding of psychological counseling centers in Italian universities. In our overly performative era, in which physical appearance and its spectacularization through social media seem to have taken over, causing significant damage to the sense of self-esteem and mental well-being of those young people who do not reach certain esthetic standards promoted by the media, our findings seem to point to a significant reversal of the trend some students would like to move toward: “You are not your physical appearance”: being satisfied in your needs means being yourself and in mental balance.

In this direction, the study highlights the importance of not only body-focused interventions, but also of understanding how the body and reflection on it can serve as a resource for self-acceptance and identity development—using the body as a starting point to foster well-being and self-esteem, potentially through mindfulness practices among young adults (cf. Brichacek et al., 2025).

Despite the strengths of this study, several limitations should be considered when interpreting the findings. First, the reliance on self-report questionnaires introduces the risk of social desirability bias, which may have led participants to underreport certain attitudes, perceptions, and behaviors, while simultaneously overreporting others. Another limitation concerns the lack of model control, as contextual or individual variables (e.g., personality traits) were not taken into account, as well as the fact that the study is cross-sectional. The study should therefore be understood as an investigation of functioning rather than causal relationships among variables, aimed at identifying which variables carry weight within a here-and-now psychological configuration. Future research should adopt a longitudinal design to more clearly capture the underlying mechanisms and developmental processes, while also considering these additional variables. Moreover, the use of multiple informants could further strengthen the validity and reliability of future investigations. Another limitation is related to the composition of the sample, which is predominantly female. Because it is a convenience sample, data were collected in courses attended mainly by women, though this was of course not the researchers’ intention. Future studies could test different models by gender and examine potential similarities and differences.

The results of our study may also not be directly generalizable to other cultures. Future studies need to investigate the factors that influence university students’ satisfaction of their basic psychological needs for relatedness, competence, and autonomy. Since few studies, especially in Nordic cultures, have explored this aspect, cross-cultural studies would be useful in this regard.

Nevertheless, the present study underscores the pivotal role of counseling services for university students. The fulfillment of developmental needs, and the consequent achievement of existential well-being, can be attained only through the promotion and preservation of sound mental health.

## Data Availability

The datasets presented in this article are not readily available because the data are part of a larger project and can be requested directly from the authors privately. Requests to access the datasets should be directed to giulio.durso@unirc.it.
